# Differential vector competence of *Ornithodoros* soft ticks for African swine fever virus: What if it involves more than just crossing organic barriers in ticks?

**DOI:** 10.1186/s13071-020-04497-1

**Published:** 2020-12-09

**Authors:** Rémi Pereira De Oliveira, Evelyne Hutet, Renaud Lancelot, Frédéric Paboeuf, Maxime Duhayon, Fernando Boinas, Adalberto A. Pérez de León, Serhii Filatov, Marie-Frédérique Le Potier, Laurence Vial

**Affiliations:** 1 UMR Animals, Health, Territories, Risks and Ecosystems (ASTRE), French Agricultural Research Center for International Development (CIRAD), Montpellier, France; 2 UMR ASTRE, CIRAD, National Research Institute for Agriculture, Food and the Environment (INRAE), University of Montpellier, Montpellier, France; 3 Swine Virology and Immunology Unit, Ploufragan-Plouzané-Niort Laboratory, French Agency for Food, Environmental and Occupational Health & Safety (ANSES), Ploufragan, France; 4 Center for Interdisciplinary Research in Animal Health (CIISA), Faculty of Veterinary Medicine, University of Lisbon, Avenida da Universidade Técnica, Lisbon, 1300-477 Portugal; 5 Knipling-Bushland U.S. Livestock Insects Research Laboratory and Veterinary Pest Genomics Center, US Department of Agriculture-Agriculture Research Service (USDA-ARS), Kerrville, TX USA; 6 National Scientific Center Institute of Experimental and Clinical Veterinary Medicine (NSC IECVM), Kharkiv, Ukraine

**Keywords:** African swine fever virus, Vector competence, Soft tick, *Ornithodoros*, Molecular biology

## Abstract

**Background:**

Several species of soft ticks in genus *Ornithodoros* are known vectors and reservoirs of African swine fever virus (ASFV). However, the underlying mechanisms of vector competence for ASFV across *Ornithodoros* species remain to be fully understood. To that end, this study compared ASFV replication and dissemination as well as virus vertical transmission to descendants between *Ornithodoros* *moubata*, *O*. *erraticus*, and *O*. *verrucosus* in relation to what is known about the ability of these soft tick species to transmit ASFV to pigs. To mimic the natural situation, a more realistic model was used where soft ticks were exposed to ASFV by allowing them to engorge on viremic pigs.

**Methods:**

*Ornithodoros moubata* ticks were infected with the ASFV strains Liv13/33 (genotype I) or Georgia2007/1 (genotype II), *O. erraticus* with OurT88/1 (genotype I) or Georgia2007/1 (genotype II), and *O. verrucosus* with Ukr12/Zapo (genotype II), resulting in five different tick–virus pairs. Quantitative PCR (qPCR) assays targeting the VP72 ASFV gene was carried out over several months on crushed ticks to study viral replication kinetics. Viral titration assays were also carried out on crushed ticks 2 months post infection to confirm virus survival in soft ticks. Ticks were dissected. and DNA was individually extracted from the following organs to study ASFV dissemination: intestine, salivary glands, and reproductive organs. DNA extracts from each organ were tested by qPCR. Lastly, larval or first nymph-stage progeny emerging from hatching eggs were tested by qPCR to assess ASFV vertical transmission.

**Results:**

Comparative analyses revealed higher rates of ASFV replication and dissemination in *O. moubata* infected with Liv13/33, while the opposite was observed for *O. erraticus* infected with Georgia2007/1 and for *O. verrucosus* with Ukr12/Zapo. Intermediate profiles were found for *O. moubata* infected with Georgia2007/1 and for *O. erraticus* with OurT88/1. Vertical transmission occurred efficiently in *O. moubata* infected with Liv13/33, and at very low rates in *O. erraticus* infected with OurT88/1.

**Conclusions:**

This study provides molecular data indicating that viral replication and dissemination in *Ornithodoros* ticks are major mechanisms underlying ASFV horizontal and vertical transmission. However, our results indicate that other determinants beyond viral replication also influence ASFV vector competence. Further research is required to fully understand this process in soft ticks.

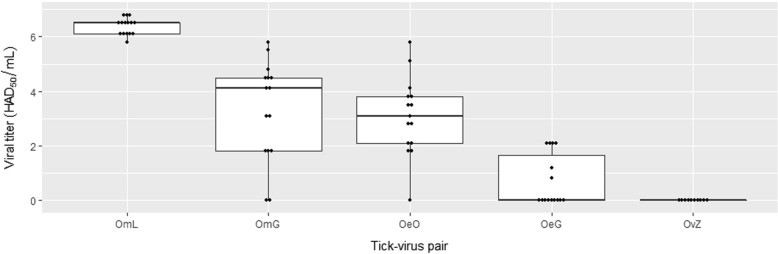

## Background

African swine fever virus (ASFV) is the etiological agent of African swine fever (ASF), a severe disease of swine that can result in up to 100% mortality in populations of domestic pigs and wild boar. Modes of ASFV transmission include pig to pig contact, contaminated food, fomites, and the bite of *Ornithodoros* soft ticks that are competent vectors [1, 2]. Vector competence is the ability of an arthropod to acquire, support replication of, and transmit a pathogen to a susceptible vertebrate host. In eastern Africa, ticks of the *O. moubata* group are known vectors and reservoirs of ASFV. In parts of Africa where ASFV is endemic, soft tick vectors enable the persistence of virus transmission within a sylvatic cycle involving warthogs, and occasionally serve as a source of infection for reemergence of the disease in domestic pigs [3]. ASFV was first introduced into Europe, specifically into Portugal and Spain, in 1957 and 1960, respectively. Soft ticks of the *O. erraticus* group were found in several southern regions of those countries where ASF has persisted [4], and vector competence of these soft ticks for local ASFV strains was demonstrated [5–7]. In the 1970s, ASF spread into western Europe, South America, and the Caribbean, but it was successfully eradicated from all those regions, with the exception of the island of Sardinia [8]. In 2007, ASF was re-introduced in Eurasia, spreading from Georgia [9] to the Russian Federation and other countries in eastern and central Europe [10, 11], and further to Asian countries, including China [12]. Quarantine, herd depopulation, and zoning are the only options to eradicate ASF outbreaks and prevent their spread because an efficient vaccine to control ASF remains to be developed [13]. This situation highlights the need to assess the vector competence (including virus reservoir role) of soft ticks established in parts of the world where ASFV is currently present or classified as a high-consequence foreign animal disease with the potential to be introduced [14].

Studies have tested the ability of different *Ornithodoros* species to transmit diverse ASFV strains. Differences in viral titer and persistence were demonstrated depending on the soft tick and virus strain combination tested [15], which highlights the diversity of factors underlying the competence of *Ornithodoros* ticks for ASFV, such as the tick species/population or virus strain. For example, in one study *O. coriaceus* was found to be able to maintain the Tengani/62 ASFV strain up to 212 days post infection (PI) [16], but in another study, *O. porcinus* was able to maintain it for only 133 days [17]. Furthermore, *O. coriaceus* was found to be able to transmit the Tengani/62 ASFV strain, but not the Uganda/61 [16, 18]. By comparison, *O. porcinus* was able to transmit both strains to domestic pigs [17]. These findings emphasize that vector competence was not only related to the tick but also to the ASFV strain. Differences in vector competence were also demonstrated in a recent study where *O. moubata* transmitted the European ASFV strain Georgia2007/1 to domestic pigs while *O. erraticus* did not [2]. However, these studies were carried out under different conditions, which makes it difficult to establish robust hypotheses regarding the discrepancies.

Few studies have investigated the replication and dissemination of ASFV within *Ornithodoros* vectors, especially its spread towards organs potentially involved in ASFV transmission. A study of *O. porcinus* infected with the Chiredzi/83/1 ASFV strain showed dissemination to the midgut, salivary glands, coxal glands, and reproductive organs [19]. Viral titers were the highest in the salivary glands and the reproductive organs, whereas virus replication was not observed in the nervous tissues of the synganglion, Malpighian tubules, and muscle, and this tick–virus pair was able to transmit ASFV to pigs [19]. Studies testing other soft tick–ASFV associations where transmission was documented also reported virus detection in different organs. For example, transmission was associated with the presence of ASFV in the salivary glands of *O. moubata* [20], hemolymph and salivary glands of *O. turicata* [16], or coxal fluid of *O. erraticus* [5]. The route of virus acquisition also influences the dynamics of infection and transmission. When infecting *O. porcinus* orally with ASFV strains Pretoriuskop/96/4/1 or Malawi Lil/20/1 using an artificial membrane feeder, virus titers measured in tick homogenates increased by tenfold in 10 days for Pretoriuskop/96/4/1, while declining 1000-fold in 2–6 days for Malawi Lil/20/1. The same Malawi Lil/20/1 ASFV strain inoculated through intrahemocoelic injection was able to replicate in *O. porcinus* [21]. Taken together, these results suggest that vector competence requires ASFV replication and dissemination in tick vectors to infect organs involved in transmission. However, gaps remain in our understanding of infection dynamics and virus dissemination in *Ornithodoros* ticks exposed to ASFV strains currently circulating in Eurasia.

To explore underlying mechanisms of ASFV vectorial transmission, in this study we compared the behavior of several strains of the virus in three *Ornithodoros* species (*O. moubata*, *O. erraticus*, and *O. verrucosus*) by measuring viral replication kinetics, viral titers at given time points, viral dissemination within ticks, and filial infection rates. The effect of the infection on ticks was also assessed. We selected these specific tick–virus pairs since the results of ASFV transmission to pigs have already been published for them [2] and could be linked to the profiles obtained in this present study. Additionally, some of these pairs were considered as positive controls that were naturally observed while the others were representative of the tick–virus associations that could possibly be encountered in the current context of ASF emergence in Europe. Our results are discussed with regards to potential determinants of vector competence in soft ticks for ASFV, related to ticks or to the virus. We also identified topics related to the competence of soft ticks as ASFV vectors requiring further investigation.

## Methods

### Virus strains

Four highly virulent ASFV strains were selected. Two of these belong to genotype II: the Georgia2007/1 strain initially isolated in 2007 from a domestic pig originating in Georgia [9], and the Ukr12/Zapo strain isolated in 2012 from a domestic pig in Ukraine [22]. Both are genetically very close [22]. The other two ASFV strains belong to genotype I: the Liv13/33 strain isolated in 1983 from *O*. *moubata* inhabiting warthog burrows in Zambia [23], and the OurT88/1 strain isolated in 1988 from *O*. *erraticus* collected from pig facilities in Portugal [5]. The Georgia2007/1, Liv13/33, and OurT88/1 strains were kindly provided by Dr. Linda Dixon (OIE reference laboratory, The Pirbright Institute, UK), and the Ukr12/Zapo strain by Dr. Carmina Gallardo (ASF European Union Reference laboratory, CISA-INIA, Valdeolmos, Spain). Viral strains used in this study were amplified on porcine alveolar macrophages, once for strains Liv13/33 and Ukr12/Zapo, and twice for strains Georgia2007/1 and OurT88/1. ASFV was diluted in RPMI medium for intramuscular inoculation of pigs as described previously [2].

### Tick species

Soft ticks of the following species reared in the laboratory were used in this study: (i) *O*. *moubata* (*s*.*s*.), as described by Bakkes et al. [24], from southern Africa (“Neuchâtel strain, maintained in the Neuchâtel University insectary for at least 20 years and reared at CIRAD Montpellier since 2008); (ii) *O. erraticus* from Portugal (“Alentejo” strain, collected from the field in 2013 and 2016 and reared at CIRAD Montpellier since that date with 1–5 generations elapsed); and (iii) *O. verrucosus* from Ukraine (collected from the field in 2014–2015 and reared at the NSC IECVM in Kharkiv, with 1 generation elapsed). All these ticks were maintained at 26 °C/80–90% relative humidity, as recommended for these species [25].

### Tick infection on viremic domestic pigs

Twenty-two Specific Pathogen-Free (SPF) pigs, split into four independent groups, were each intramuscularly inoculated with a 10^4^ hemadsorbing dose 50% (HAD_50_) of one of the different viral strains listed above. When the pigs became viremic between 3 and 4 days post inoculation, ticks were infected by feeding on them. Petri dishes containing ticks enclosed with a mosquito net were kept on each pig’s lower abdomen with a bandage to allow tick feeding through the mesh for 3 h. On the day of tick exposure to ASFV via feeding, pig viremia was quantified by titration; viremia was found to range from 10^7.5^ HAD_50_ to 10^8.25^ HAD_50_/ml. Five tick–virus combinations were obtained: *O. moubata*–Liv13/33 (OmL), *O. moubata*–Georgia2007/1 (OmG), *O. erraticus*–OurT88/1 (OeO), *O. erraticus*–Georgia2007/1 (OeG), and *O. verrucosus*–Ukr12/Zapo (OvZ).

### Tick sample processing

**Fig. 1:**
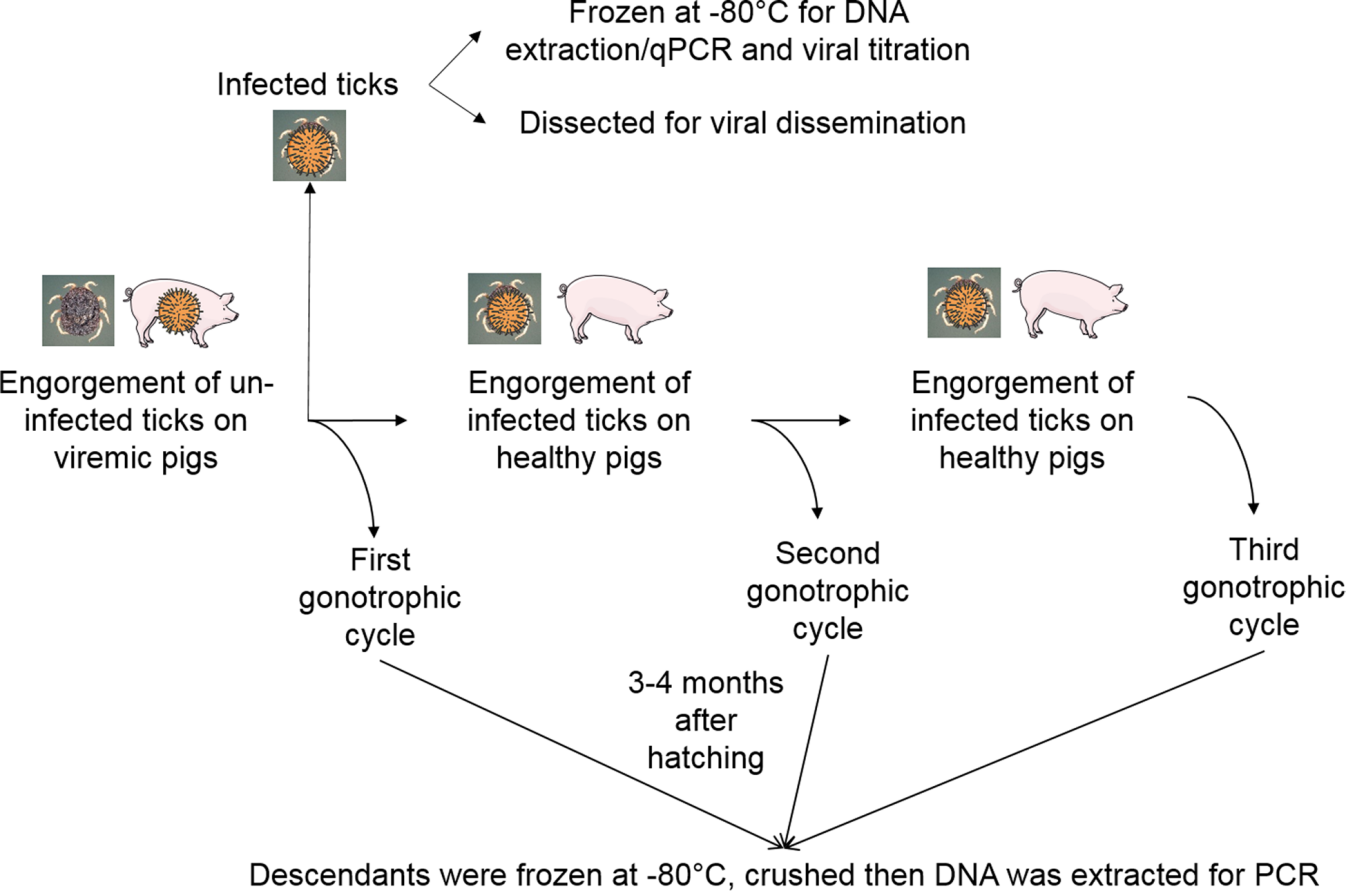
Depiction of the experimental design used to study the development of African swine fever virus (ASFV) in the infected *Ornithodoros* soft ticks

After the infectious blood meal, ticks were sorted into pools dedicated to these analyses: (i) monitoring of ASFV replication in ticks using quantitative real-time PCR (qPCR) on tick homogenates; (ii) estimation of the viral load in ticks using virus titration; (iii) localization of the virus in ticks using qPCR on internal organs after dissection; and (iv) assessment of ASFV vertical transmission by analyzing descendants of all the tick–virus pairs by qPCR. Localization assays could not be carried out for OvZ, due to a lack of material. The number and the stage of ticks used for assays (i) and (ii) are shown in Additional file 1: Table S1. The overall experimental design is shown in Fig. 1. Ticks were individually crushed in phosphate buffered saline solution (PBS) as previously described [2] and stored at − 80 °C for further analysis. For the kinetic study, tick homogenates were assayed at 0, 1, 2, and 3 months PI for all the tick–virus pairs. Additional assays were performed at 8, 10, and 12 months PI for OeO and OeG, and at 13 months PI for OmG and OvZ. No additional time points were added for OmL due to the high mortality in this tick–virus pair (see Results section). For virus titration, the same tick homogenates were assayed 2 months PI for all the tick–virus pairs. Additional assays were carried out at 3 months PI for OmL, at 8 months PI for OeO and OeG, and at 13 months PI for OmG. The measure at 0 month PI determines the initial amount of virus ingested by individual ticks.

For ASFV localization, ticks were dissected using a sterile scalpel blade and surgical pliers to obtain samples of intestine, salivary glands, and reproductive organs. Dissected organs were stored at − 80 °C in 200 µl of PBS until tested. The dissections of specimens representing all tick–virus pairs were carried out at 10 months PI, and at 3 months PI for OeO and OeG as an additional time point. At 10 months PI, five male ticks and five female ticks were dissected for OeG and OmG, nine females and one male for OeO, and three females and two males for OmL. At 3 months PI, five female and five male ticks were dissected for OeG and OeO.

To test vertical transmission to descendants, i.e., filial infection, we used the first progeny cohort produced following the initial feeding on viremic pigs by female ticks sampled for this purpose. Second and third cohorts of progeny were obtained after the females fed once or twice more on healthy pigs. The third cohort was only available for OmL, OmG, and OeG. Descendants from the first gonotrophic cycle were not kept to be tested for OeO, OeG, and OvZ. The third cohort of progeny was unavailable for OeO and OvZ because these ticks were not engorged repeatedly. Only the second cohort was available for these two tick–virus combinations. Tick progeny were tested for ASFV soon after egg hatching, namely larvae for *O. erraticus* and *O. verrucosus*, and the first nymphal stage for *O. moubata*, since larvae of this latter species molt directly to the next developmental stage without blood-feeding [26]. Descendants were frozen at − 80 °C between 3 and 4 months after hatching and stored at − 80 °C until processed. Descendants of OmL and OmG were crushed in 1 ml of PBS, whereas OeO, OeG and OvZ were crushed in 200 µl of PBS due to their smaller size. Initial screenings were done using pools of 20 ticks crushed together. Individual detection was carried out only on a few individuals (8–32) for some tick–virus pairs that did not transmit ASFV vertically. For tick–virus pairs testing positive in the initial screening, 38 to 206 descendants were tested individually to estimate filial infection rates.

As individuals were kept for several months to monitor ASFV kinetics in ticks and ASFV vertical transmission, it was possible to detect potential extra tick mortality due to ASFV infection. However, we did not check tick survival at regular and predefined time points. Thus, an estimated mortality rate for each tick–virus pair was obtained at 16.5 months PI when the experiment ended. Mortality rates were considered to be high when they reached 5%, which is the maximum natural mortality recorded for our *O. moubata* tick colony in the absence of treatment [27]. The same threshold was applied to the other *Ornithodoros* species tested because reference values were unavailable in the scientific literature.

### Viral genome detection in whole ticks and tick organs

From ticks used to monitor viral replication or from the progeny used to test vertical transmission, DNA was extracted from 200 µl of each crushed tick supernatant using the High Pure PCR Template Preparation Kit (Roche Life Science, Penzberg, Germany). Primers and probes detecting the VP72 ASFV gene and the tick beta-actin gene were used for duplex qPCR as described previously [2]. For absolute quantification, a plasmid encoding the VP72 and tick beta-actin genes was used at different dilutions to obtain a standard curve and determine the number of gene copies in each sample (Additional file 2: Figure S1).

The qPCR described above was used to assay dissected tick organs. Organs were lysed using tissue lysis buffer according to the manufacturer’s procedure (High Pure PCR Template Preparation Kit; Roche Life Science); DNA was extracted using the same kit (Roche Life Science). As we did not monitor ASFV replication in organs and just needed an estimation of the genome viral load in organs at a given time, amplification results were only expressed as the quantification cycle (Cq), i.e., the number of amplification cycles required for the fluorescent signal to cross the detection threshold. A low Cq indicates a large quantity of DNA, while a high Cq indicates a small quantity of DNA.

### Virus titration

Tick homogenates were centrifuged at 10,000* g* for 5 min. The supernatant was serially diluted in RPMI for viral titration in porcine alveolar macrophages, and pig erythrocytes were added the next day as described previously [28]. Viral titers were expressed as HAD_50_/ml.

### Statistical analysis

Experimental data were analyzed using the R statistical package in the RStudio environment (R Foundation for Statistical Computing, Vienna, Austria). Throughout the analysis, we used α = 0.05.

To analyze viral replication, the ratio of the VP72 ASFV gene and tick beta-actin gene copy numbers was calculated assuming that: (i) both genes were present in a single copy per genome; (ii) the ratio between VP72 and beta-actin provided the number of ASFV genomes relative to tick genomes, thus correcting for tick size. The number of beta-actin copies in some samples (1 individual in OeG and 7 in OvZ at 0 month PI) was much lower than for other ticks of the same species and stage, which resulted in a significant but abnormal increase in ratios. These ticks may have died before being frozen, which increased the risks of DNA degradation. The outliers with ratios > 300 were arbitrarily excluded from the analysis.

However, ratios remained very different between individuals, and also between tick–virus pairs. To put all individuals on the same scale and account for ratios equal to 0 (non-infected ticks), each ratio *x* was firstly transformed into log (1 + *x*). Preliminary exploratory data analysis revealed each tick–virus pair had a different mean and dispersion. To facilitate the analysis, data of each tick–virus pair were secondly centered and scaled on their own mean and standard deviation according to the formula:

$$\frac{{\log \left( {1 + x} \right) - mean\left[ {\log \left( {1 + x} \right)} \right]}}{{sd\left[ {\log \left( {1 + x} \right)} \right] }}$$

Thus, after this transformation, all tick–virus pairs had the same mean (= 0) and the same standard deviation (= 1).

A generalized least squares model model was used to account for heteroscedasticity in residuals for each combination of tick–virus × PI time. This model provided a slope time coefficient for replication kinetics for each tick–virus pair. These time coefficients were compared to the mean to determine whether differences were significant. As no assumption was made on the effect of soft tick sex/stage on vector competence for ASFV, and our initial exploratory data analysis did not detect differences, we grouped sexes and stages together for the overall analysis.

To compare viral titer 2 months PI and to account for the small number of individuals per tick–virus pair (≤ 15), we used non-parametric tests. A Kruskal-Wallis test was used first to detect overall differences between all tick–virus pairs. Then, pairwise Wilcoxon tests were used to determine which tick–virus pairs had different viral titer.

To analyze viral dissemination in ticks, Cq was categorized into four classes: (i) < 25 Cq, corresponding to a high load of the VP72 gene; (ii) 25–35 Cq, for an intermediate load of the VP72 gene; (iii) 35–45 Cq, for a low load of the VP72 gene; and, (iv) Negative, for negative samples. To determine if the tick–virus pair or the nature of the organ had an effect on viral spread in ticks, an ordinal logistic regression model was used, with the proportion of ticks for each modality as the explanatory variable. OeG was chosen as the reference class for the “tick–virus pair” variable, and salivary glands were the reference for the “organs” variable. A likelihood ratio test was used to assess the effect of variables on the deviance of the model.

A multiple correspondence analysis was applied to detect correlations between the different biological parameters measured in ticks, and the tick–virus pairs that were characterized by distinct abilities to transmit ASFV to pigs. This allowed the data to be summarized and projected on a few orthogonal axes chosen to maximize the projected variance. Results were interpreted in terms of Euclidean distance between the categories of virus replication, viral titer, and dissemination in ticks for ASFV vector competence. Table 1 summarizes how biological parameters were arranged in three to five categories and linked to four tick–virus pair classes (OmL, OmG, OeO and OeG) for the analysis.

**Table 1:** Description of biological parameters used for correspondence analysis

Biological parameter	Name of biological parameter used in analysis	Method used to obtain results	Categories
Intestine	intestine_neg	qPCR	Negative ASFV PCR
	intestine_1		Cq between 45 and 35
	intestine_2		Cq between 35 and 25
	intestine_3		Cq <r 25
Salivary glands	sg_neg	qPCR	Negative ASFV PCR
	sg_1		Cq between 45 and 35
	sg_2		Cq between 35 and 25
	sg_3		Cq < 25
Reproductive organs	ro_neg	qPCR	Negative ASFV PCR
	ro_1		Cq between 45 and 35
	ro_2		Cqt between 35 and 25
	ro_3		Cq < 25
Viral titer	titer_neg	Viral titration	Viral titer between 10^0^ and 10^2^
	titer_1		Viral titer between 10^2^ and 10^4^
	titer_2		Viral titer between 10^4^ and 10^6^
	titer_3		Viral titer between 10^0^ and 10^2^
	titer_4		Viral titer over 10^6^
Replication rates	up_qpcr	qPCR	Significant increase in viral replication
	down_qpcr		Significant decrease in viral replication
	no_qpcr		No significant variation in viral replication

ASFV, African swine fever virus; Cq, quantitative cycle (PCR); qPCR quantitative PCR

### Ethics statement

Animal experiments were authorized by the French Ministry for Research (project No. 2017062615498464) and approved by the national ethics committee (Authorization No. 11/07/17-3).

## Results

### Mortality in ASFV infected ticks

The tick–virus pairs OmL and OmG showed very high mortality rates (100 and 73%, respectively), while those of OeG, OeO, and OvZ survived to ASFV infection and showed mortality rates of < 5% (2.8, 2.1, and 0%, respectively). Deaths in OmL and OmG primarily occurred in ticks dedicated to vertical transmission trials after their second and third blood meals on naive pigs. Ticks used to monitor ASFV replication, viral load, and ASFV dissemination had not been fed since their initial infective blood meal and did not show increased mortality; consequently, mortality was not taken into account in the analysis of these biological parameters.

### ASFV replication in ticks

**Fig. 2:**
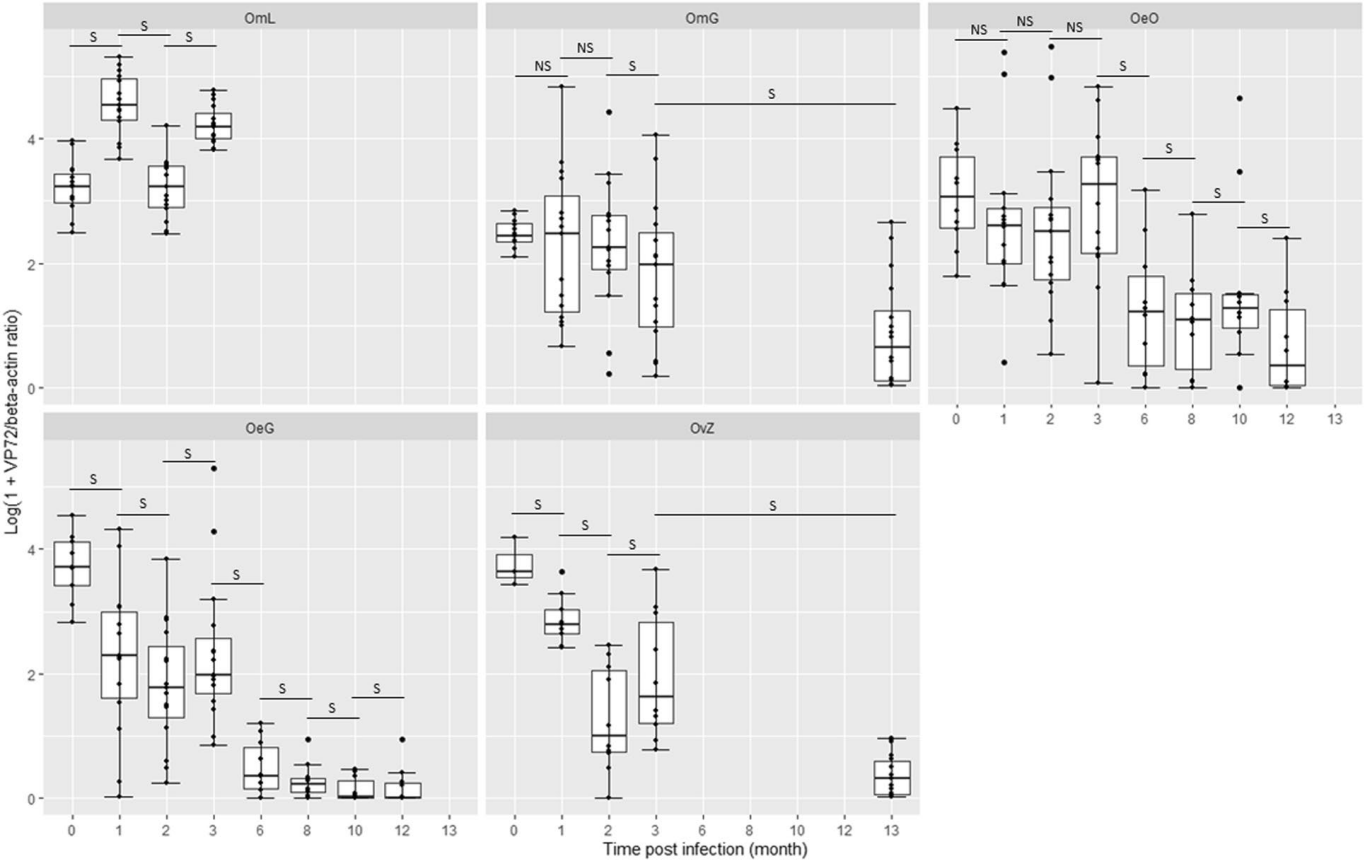
Boxplot of ASFV replication rate for all tick–virus combinations tested. Replication rate is log (1 + VP72/beta-actin). Only data with ratios < 300 were used to calculate the log (1 + VP72/beta-actin). Tick–virus combinations:* OmL* *Ornithodoros moubata*–Liv13/33,* OmG* *O. moubata*–Georgia2007/1,* OeO* *O. erraticus*–OurT88/1,* OeG* *O. erraticus*–Georgia2007/1,* OvZ* *O. verrucosus*–Ukr12/Zapo.* S* Statistically significant difference (*P* < 0.05),* NS* not statistically significant (*P* > 0.05)

Replication rates increased significantly for OmL between 0 and 1 month PI, as well as between 2 and 3 months PI (*P* < 0.05 for both), but decreased significantly between 1 and 2 months (*P* < 0.05) (Fig. 2). Conversely, the ratios decreased significantly from 0 to 12 months PI for OeG (*P* value < 0.05) and from 0 to 13 months PI for OvZ (*P* < 0.05) (Fig. 2). For OeO and OmG, the ratios did not vary significantly in the first 2 months and then decreased significantly from 3 to 12 months PI for OeO (*P* value < 0.05) and between 3 and 13 months for OmG (*P* < 0.05) (Fig. 2).

### Viral load in ticks

**Fig. 3:**
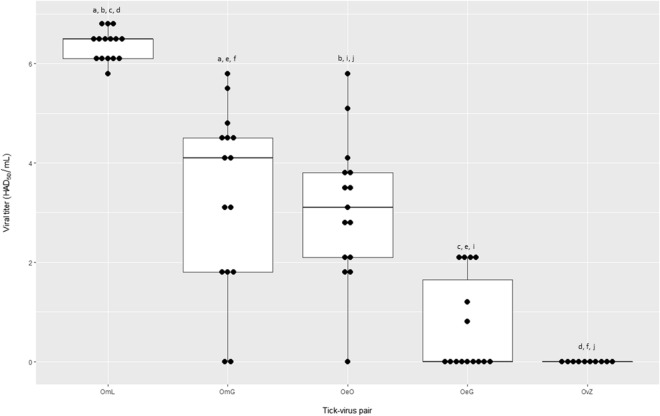
Boxplot of ASVF titers 2 months post infection for all of the *Ornithodoros* tick–virus combinations tested. Each black dot corresponds to one tick. Abbreviations for tick–virus combinations tested are as given in Fig. 2 caption. Different lowercase letters indicate significant statistical differences (*P* < 0.05) in viral titer between tick–virus combinations.* HAD*_*50*_ Hemadsorbing dose 50%

At 2 months PI, ASFV was isolated from 6/15 ticks in the tick–virus combination OeG, from 14/15 ticks in OeO, from 13/15 ticks in OmG, and from 15/15 in OmL; no viral particles were isolated from ticks in OvZ (0/10). With a* P* value of 3.69e-14, the Kruskal–Wallis test indicated significant differences in virus titer between the tick–virus pairs. Using the pairwise Wilcoxon test, viral titers were not significantly different between the tick–virus combination OeG and OvZ (*P* = 0.05687) or between OeO and OmG (*P* = 0.54571), with zero to low viral loads in OvZ and OeG (< 10^2.1^ HAD_50_/ml) and intermediate to high viral loads in OeO and OmG (< 10^5.8^ HAD_50_/ml) (Fig. 3). The differences were significant between other tick–virus combinations (*P* < 0.05) (Fig. 3). Ticks in tick–virus combination OmL displayed a significantly much higher viral load than in those the other combination groups (> 10^5.8^ HAD_50_/mL) (*P* value < 0.05), with particularly low variance between observations (Fig. 3). At 8 months PI, ASFV could still be isolated from 1/10 ticks in the OeG tick–virus combination (10^2.1^ HAD_50_/ml), and from 3/10 ticks in OeO (10^2.1^, 10^2.8^, and 10^3.5^ HAD_50_/ml, respectively), with the viral loads seemingly decreasing in OeO. At 13 months PI, ASFV particles were still isolated from 7/16 ticks in OmG (10^2.1^–10^5.5^ HAD_50_/ml), with also an apparent decrease in viral load, although the difference was not statistically tested. It was not possible to test the long-term persistence of ASFV live particles for the tick–virus combination OmL due to the high mortality observed in OmL ticks as soon as they blood-fed and the necessity to keep the last surviving ticks for the horizontal and vertical transmission trials. At the last titration assay at 3 months PI, ASFV was isolated from 15/15 ticks in OmL, with viral loads similar to those observed at 2 months PI (10^5.5^–^6.5^ HAD_50_/ml).

### ASFV dissemination in ticks

**Fig. 4:**
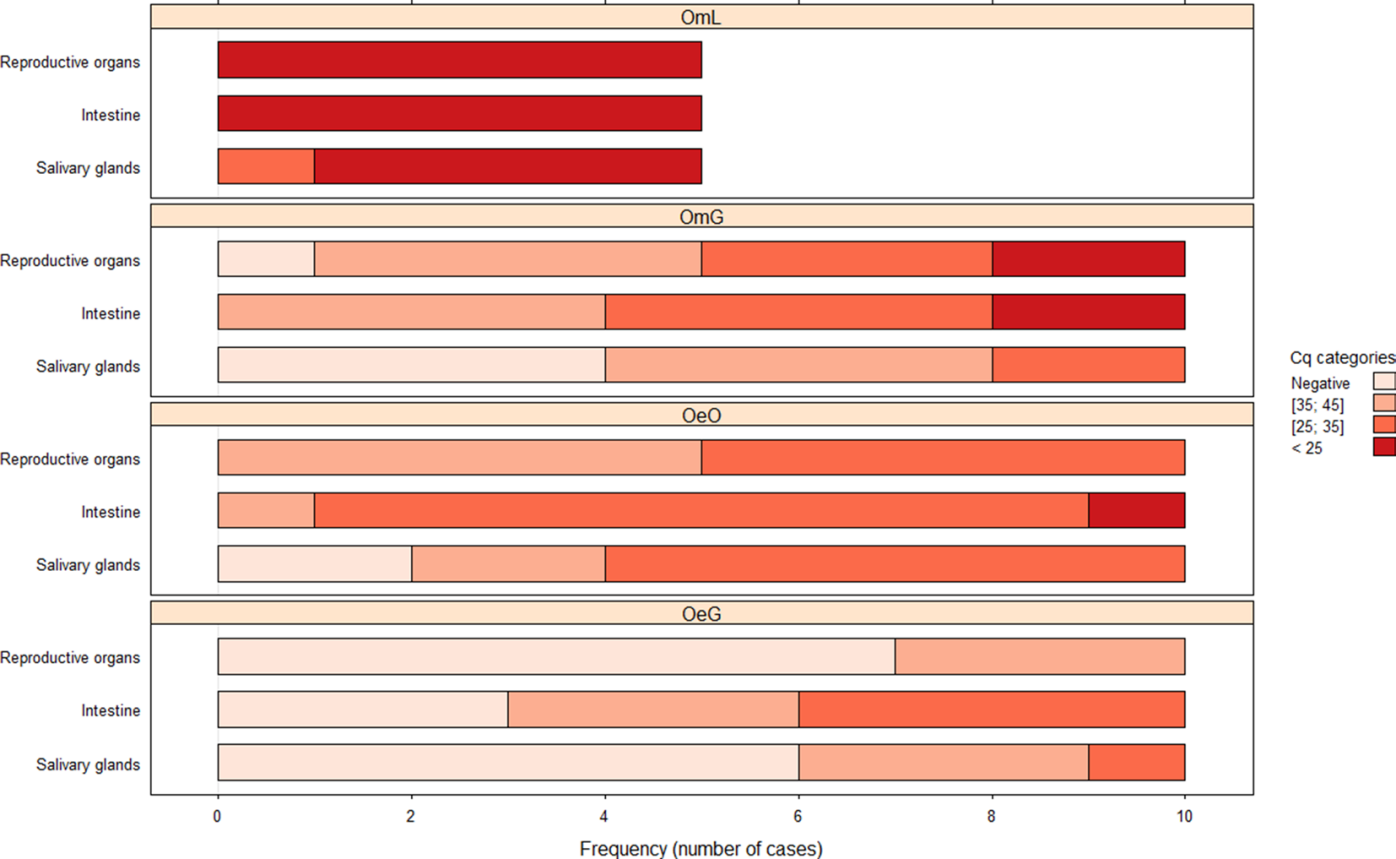
ASFV localization in organs of soft ticks 10 months post-infection. PCR results, expressed as the quantification cycle (Cq) were split into four categories: (i) Negative organs, when no ASFV genome was detected; (ii) 35–45 Cq, for organs with a low load; (iii) 25–35 Cq, for an intermediate load; and, (iv) < 25 Cq, for a high ASFV genome load. Three organs were analyzed: reproductive organs, intestine, and salivary glands. The abbreviations for tick–virus combinations tested are as given in Fig. 2 caption

**Fig. 5:**
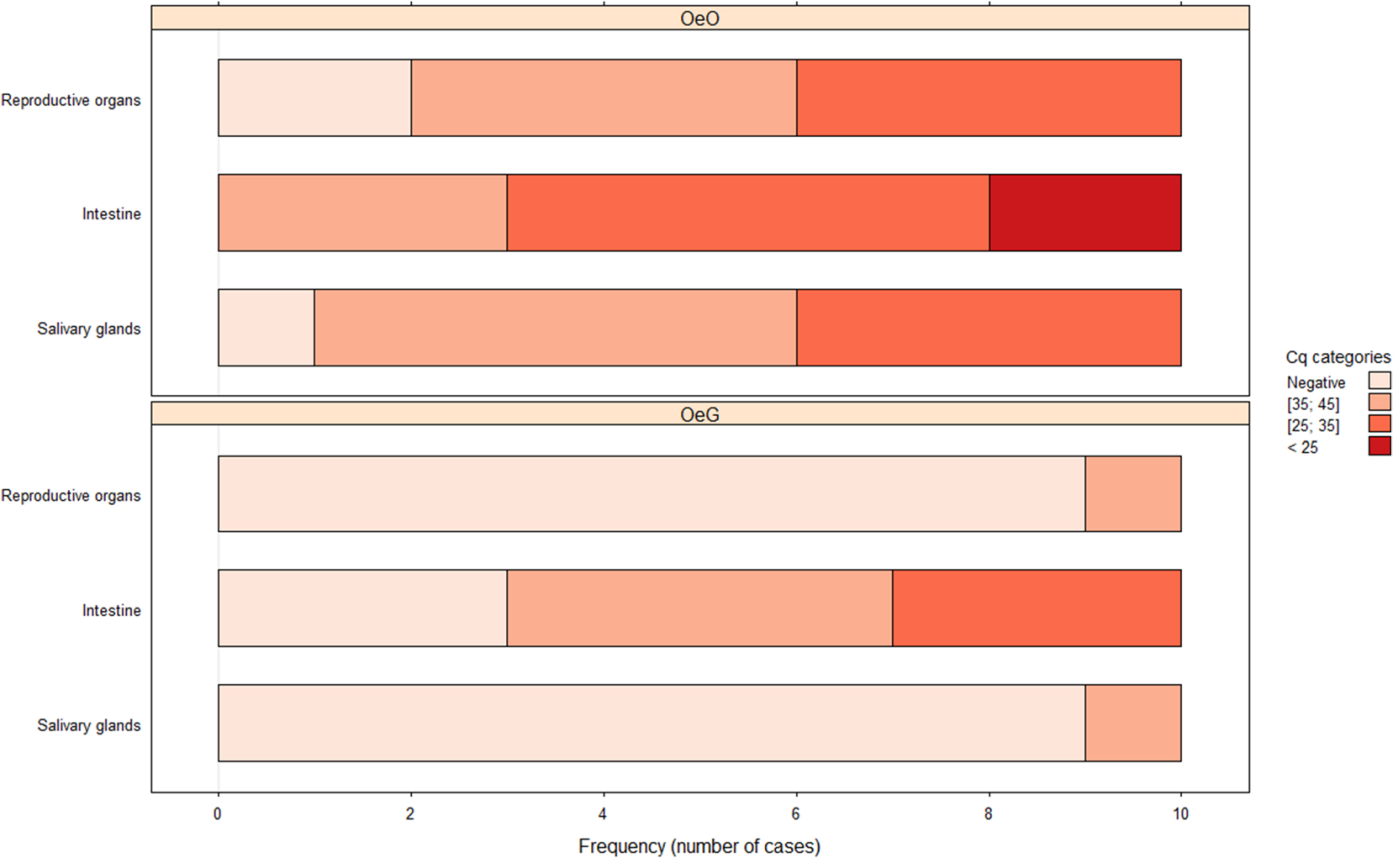
ASFV localization in infected soft ticks 3 months post-infection. PCR results, expressed as Cq) were split into four categories: (i) Negative organs, when no ASFV genome was detected; (ii) 35–45 Cq, for organs with a low load; (iii) 25–35 Cq, for an intermediate load; and (iv) < 25 Cq for a high ASFV genome load. Three organs were analyzed: reproductive organs, intestine, and salivary glands. The abbreviations for tick–virus combinations tested are as given in Fig. 2 caption

The results of ASFV DNA detection in tick organs 10 months PI are shown in Fig. 4. The results for the tick–virus combinations OeO and OeG 3 months PI are shown in Fig. 5. At 10 months PI, the ordinal logistic model showed a significant effect of the tick–virus combination (*P* < 2.2e-16) and the nature of the organ (*P* = 0.000729) on Cq levels. Most of the tested organs in the tick– virus combination OmL showed the highest Cq levels, whereas the other tick–virus pairs showed a wide range of Cq levels in all organs, including also negative results (Fig. 4). OeG was the only tick–virus combination for which 100% of intestine samples were positive at 10 months PI (Fig. 4). However, as the first barrier encountered by ASFV after an infectious blood meal, the intestine remained the most frequently infected organ in all tick–virus pairs. Regarding the other organs, infected salivary glands were recorded in OmL (100%), OeO (80%), OmG (60%), and then OeG (40%), the latter being the tick–virus combination with the lowest number of infected salivary glands at 10 months PI (Fig. 4). While the OmL, OmG and OeO tick–virus combinations showed 90–100% positivity of reproductive organs, OeG showed only 30% of infected reproductive organs at 10 months PI (Fig. 4). Similar results were observed at 3 months PI for the OeO and OeG tick–virus combinations (Fig. 5).

### Filial infection with ASFV

All the tested progeny of OmG, OeG and OvZ were ASFV negative. For OeO, only a single specimen from the second gonotrophic cycle was positive. This corresponded to a filial infection rate of 0.48% (Table 2). For OmL, positive descendants were detected in every tested cohort. The proportion of infected individuals increased from 9.4% to 65.8% and 69.4% for the first, second, and third gonotrophic cycles, respectively) (Table 2).

**Table 2:** Vertical African swine fever virus transmission in each tick–virus combination tested

Gonotrophic cycle	tick–virus pair^a^	Treatment	Filial infection rates^b^ (%)
First	OmL	Individual	10/106 (9.4%)
		Pool	6/10 (60%)
	OmG	Individual	0/8
		Pool	0/10
Second	OmL	Individual	25/38 (65.8%)
	OmG	Individual	0/32
		Pool	0/5
	OeO	Individual	1/206 (0.48%)
	OeG	Individual	0/30
		Pool	0/10
	OvZ	Pool	0/1
Third	OmL	Individual	25/36 (69.4%)
	OmG	Individual	0/8
		Pool	0/4
	OeG	Individual	0/9
		Pool	0/10

^a^* OmL*, *Ornithodoros moubata*–Liv13/33;* OmG*, *O. moubata*–Georgia2007/1;* OeO*, *O. erraticus*–OurT88/1;* OeG*, *O. erraticus*–Georgia2007/1;* OvZ*, *O. verrucosus*–Ukr12/Zapo

^b^Positive individual ticks or pools of 20 ticks (Pool) each/total tested

### Correlation between biological parameters measured in ticks and transmission ability

**Fig. 6:**
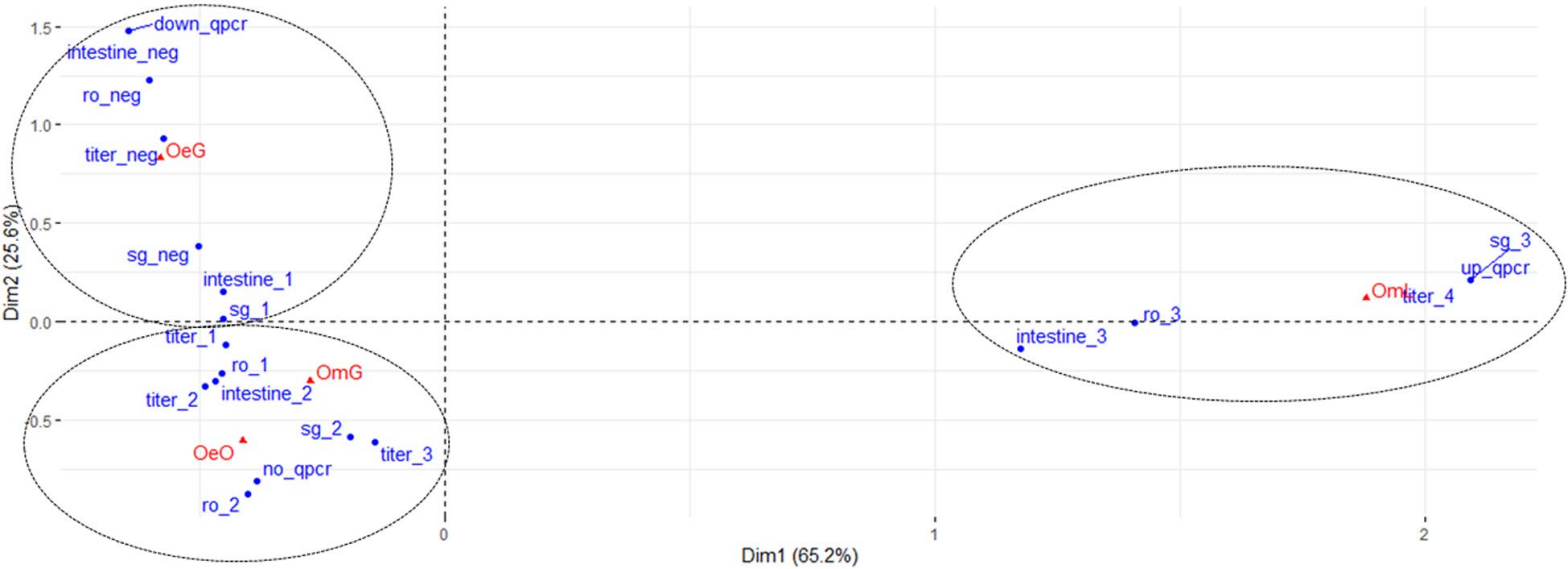
Bi-dimensional plot showing the correspondence analysis of multiple variables and different tick–virus combinations.* Dim* Dimension. See section Materials and Methods for details. The third axis is not represented in the bi-dimensional plot

Multiple correspondence analysis defined two main synthetic axes explaining 65.2% (dimension 1) and 25.6% (dimension 2) of data variance, respectively. Dimension 3 explained only 9.2% of data variance. The tick–virus combination OmL, characterized by a full transmission capacity, contributed mostly to dimension 1, and OeG, which was not able to transmit in any cases, greatly contributed to dimension 2. The intermediate virus–tick combination OmG, which only transmitted horizontally, mainly contributed to dimension 3. OeO, which rarely transmitted to its descendants was divided between dimensions 2 and 3. Increased ASFV replication rates, high viral titers, and high Cq values in organs mainly contributed to the first axis and were all correlated to tick–virus combination OmL (Fig. 6). Conversely, decreased ASFV replication rates, low to zero viral titers, and high Cq values in organs mainly contributed to axis 2 and were correlated to OeG (Fig. 6). OmG and OeO were associated with intermediate ASFV replication and dissemination patterns, which contributed to both the second and third axes (Fig. 6).

## Discussion

This study highlighted differences in the kinetics of ASFV replication and dissemination inside *Ornithodoros* vectors. The results are in agreement with those reported previously showing variation in virus titers and persistence of infection across *Ornithodoros* species that is dependent on the ASFV strain used [15]. On the one side of the variability spectrum we observed that the OmL tick–virus combination resulted in ASFV transmitted horizontally and vertically with increasing replication, high viral titers, and efficient virus dissemination to the internal organs. On the other side of the spectrum, ASFV in the OeG tick–virus combination was not transmitted to pigs or to the *O*. *erraticus* progeny, virus was cleared over time, and ticks yielded low viral titers with poor organ dissemination. Interestingly, the other two tick–ASFV combinations displayed intermediate profiles, with OmG transmitting ASFV only horizontally and OeO transmitting only vertically, but with a very low efficiency rate (a single positive tick out of 206 tested). Both of these latter tick–virus combinations showed similar ASFV replication rates, as well as similar viral titers 2 months PI and similar dissemination efficiencies.

The results presented here suggest that events other than the infection of internal organs are required for *Ornithodoros* ticks to transmit ASFV. In a previous study on ASFV horizontal transmission, we showed that the OmG tick–virus combination resulted in virus transmission when 30 ticks bit a pig at a single time [2], whereas transmission failed when multiple tick challenges were carried out using 15 ticks each time. We thus hypothesized that the proportion of infected soft ticks, and thus the proportion of ticks with infected internal organs, is important factor in the transmission success. Quantitative aspects related to bioactive salivary gland factors may also play a role in the competence of *Ornithodoros* ticks as ASFV vectors [29]. Furthermore, we showed, using correspondence analysis, that ASFV replication in ticks, as well as the resulting viral loads in particular internal organs, are additional essential factors for explaining transmission success. However, it must be noted that we did not determine viral dissemination in the OvZ tick–virus combination as an insufficient number of ticks were available.

The observation that while *O. moubata* transmitted ASFV to pigs efficiently they also perished from viral infection suggests that *Ornithodoros* species differ in their capacity to control ASFV infection. However, this excess mortality does not alter the vector competence of *O. moubata* as it only occurred after the second blood meal when ASFV was transmitted horizontally and/or vertically. As mentioned before [15], this stresses the need for research to enhance our understanding of the molecular basis of the interaction between ASFV and soft ticks. In the less effective tick–virus pairs, OeG and OvZ, which did not die from infection, ASFV replication decreased considerably, especially by 2 months PI, which indicates a gradual clearance of the virus from the ticks. However, in another study using the Georgia2007/1 strain and *O. erraticus* ticks, ASFV replicated very efficiently [30]. The main difference between the results of that and our studies is that the ticks used by Diaz et al. [30] were artificially engorged on blood in the presence of antibiotics and antifungals. This treatment may have directly modified the integrity of the tick midgut, favoring the crossing of ASFV into the hemocoel and therefore bypassing midgut replication (known as the “leaky-midgut” phenomenon) [31]. Another hypothesis is that the presence of such substances in the blood meal could have affected the results because antibiotics are known to alter the microbiome and the metabolism of ticks [27].

Microbiome modifications in arthropod vectors are known to affect the replication of pathogens, including viruses [32–35]. Interestingly, when they were reared in Neuchâtel before being transferred to CIRAD, *O. moubata* were maintained on blood treated with antibiotics for several years. A study using *Aedes aegypti* showed shown that a modification of the midgut microbiome had an impact on the basal immune response of mosquitoes by changing the expression of genes involved in the immune response, such as antimicrobial peptides, which in turn impacted Dengue virus replication [34]. In the hard ticks, *Ixodes ricinus* and *Haemaphysalis longicornis,* defensins have been shown to have an antimicrobial effect on Gram-positive bacteria [36, 37]. In the soft tick *Ornithodoros moubata*, a challenge with *E. coli* upregulated expression of the gene coding for defensins A and B in the midgut [38]. It was also reported that soft tick engorgement changed the expression of immune genes, such as the genes coding for antimicrobial peptides, in different soft ticks species (*O. moubata*, *O. erraticus* and *O. turricata*) [38–43]; however, the antiviral effect of this changed expression needs to be tested. Among the large panel of immune responses described in arthropods against transmitted pathogens [44], RNA interference is an important immune response against virus infection, having an impact on vector competence [45]. The authors of a recent study suggested that ASFV-like integrated elements coding for small RNA in *O. moubata* could serve to protect ticks from ASFV infection through the RNA interference mechanism [46]. More specifically, the alignment of these small RNAs on the full ASFV genome showed more than 500 small RNAs that matched with ASFV genomes from genotype II (like Georgia2007/1), while there were fewer than 20 matching small RNAs with several ASFV genomes from genotype I. These results might also be an explanation for the differences in ASFV kinetics and mortality rates observed in *O. moubata* ticks infected with Liv13/33 from genotype I (OmL)* versus* *O. moubata* infected with Georgia2007/1 from genotype II (OmG).

Previous studies revealed that some ASFV genes, such as those of the Multigen family 360 (MGF-360) and CD2v protein, could be involved in viral replication in ticks, with the deletion of these genes having a negative impact on the efficiency of virus replication in the soft ticks *O*. *porcinus* and *O*. *erraticus*, but not blocking it [47, 48]. Molecular analysis of the viral strains Liv13/33 [49] and Georgia2007/1 [50] used in our experiments showed the existence of these genes in both strains. The genomes of OurT88/1 and Ukr12/Zapo remain unknown, which makes the comparison with these ASFV strains difficult. However, ASFV replication differed between the tick–virus combinations OmL and OmG, and even the presence of these ASFV genes might not be sufficient to explain differences in ASFV replication in these tick–virus combinations, which raises the possibility that other as yet undetermined viral genes could affect the ability of ASFV to replicate in soft ticks. Other genes may be involved in ASFV replication in ticks. Another comparison between Liv13/33 and Georgia2007/1 highlighted the presence of the X64R gene in Liv13/33 but not in Georgia2007/1, which suggests that this gene could be implicated in viral replication (http://asfvdb.popgenetics.net/) [49]. The X64R gene is also present in most, but not all, strains of genotype I, but has never been reported for genotype II, including the Georgia2007/1 strain (http://asfvdb.popgenetics.net/). Further research is required to test the hypothesis that the presence of such genes might contribute to the ability of the tick–virus combinations OmL and OeO to transmit ASFV vertically, contrary to OmG, OeG, and OvZ as observed in our study. Notably, vertical transmission has been reported with ASFV strains from genotype I [16, 51] and in two cases with strains from genotype XX and an undefined genotype [21, 52]. Strains from genotype II and X failed to be transmitted vertically by ticks [18, 53], and in one case transmission also failed with the L60 strain from genotype I [18], although this latter strain has the X64R gene. However, a complete sequence was not available for all the ASFV strains used in these vertical transmission studies, which limits genome comparisons. The possible role of certain viral genes in replication in soft ticks highlights the complexity of ASFV–*Ornithodoros* interactions underlying vector competence [54].

Noteworthy is the vertical transmission of Liv13/33 in the OmL virus–tick combination to the first progeny after the infectious blood meal. By comparison, Rennie et al. [51] observed vertical transmission in the second gonotrophic cycle with the same soft tick–ASFV combination. To the best of our knowledge, this is the first time that this transmission pattern is described with ASFV [18, 51–53, 55, 56] or any other soft tick-borne pathogen such as *Borrelia duttoni* [26]. *Ornithodoros moubata* ovogenesis begins 4–5 days after the blood meal, allowing the acquisition of an impermeable membrane that may prevent the infection of eggs, as demonstrated for *Borrelia duttoni* [26]. Soft ticks lay eggs about 15 days after a blood meal [25]. As it takes an average of 2–3 weeks for ASFV to disseminate inside soft ticks and reach the reproductive organs after the infectious blood meal [19], it seems unlikely for the eggs of the first gonotrophic cycle to become infected. As we ensured that our *O. moubata* ticks were free of AFSV before experimentation, we surmise that ASFV dissemination inside soft ticks resulted in the infection of the latest maturated portion in the egg batch. This is congruent with the observation that < 10% of descendants in the first gonotrophic cycle were infected while > 65% were positive in the second and third cycles. Differences between our study and the experiment by Rennie et al. [51] include the method of engorgement and the initial virus titer to infect ticks. Under our experimental conditions, *Ornithodoros* ticks were infected with ASFV by taking a blood meal on viremic pigs with virus titers of between 10^7.5^ and 10^8.25^ HAD_50_/ml while Rennie et al. [51] infected ticks with an artificial feeding system using a mix of uninfected blood and viremic blood with a final titer of 10^5^ HAD_50_/ml. The initial dose might have affected the speed of ASFV dissemination in the ticks and thus the infection of the reproductive organs and transmission to the eggs. In other arbovirus-vector systems, the initial dose is known to affect the speed of virus spread, as shown, for example, with Bluetongue virus in *Culicoides sonorensis* [57] and for Zika virus in *Aedes aegypti* [58]. Equally plausible is a higher susceptibility for ASFV infection in the *O. moubata* we used that have been reared under laboratory conditions for ~ 25 years relative to the conspecific yet genetically distinct ticks used by Rennie et al. [51]. Keeping *O. moubata* as a closed colony in the laboratory for that period of time may have rendered the ticks highly inbred with fixed alleles, and a poor microbiome receiving only blood from healthy animals would limit exposure to a diverse micro-organisms, resulting in a poorly stimulated or even immunologically naive immune system compared to ticks collected directly from the field [59]. It is likely that in the wild, ticks are probably exposed to ASFV multiple times through blood-feeding during their development cycle, which can range from 6 months to several years to complete, depending on host availability and environmental conditions [25]. It is possible that constant exposure to environmental microbes shapes immunological maturity, training the innate immune system and phenotypic susceptibility to a local microbe, as well as inducing trained immunity, all factors providing cross-protection against other pathogens [59].

The interplay between genetic and environmental factors influences how the competence of arthropods as vectors of infectious agents evolves. This needs to be taken into consideration when trying to extrapolate results from laboratory experiments to try to explain what happens in the field. For example, in a previous study, two laboratory colonies of *O*. *turicata* established using ticks collected at different times and from different zones did not display the same results of vector competence for ASFV, as one was able to transmit the virus to pigs while the other one was not [16]. In this context, the genetic evolution hypothesis may explain why, under our experimental conditions, the tick–virus combination OeO did not transmit ASFV to pigs [2], but on rare occasions did transmit the virus to its descendants. In studies conducted 20 years previously, the same tick species was able to transmit the same strain (OurT88/1) horizontally [5]. The *O. erraticus* ticks used in these studies were from colonies established with field-collected specimens from the same region, but not the same pig farms and thus they represented different populations. In addition to space, collection times also differed. The colony sourcing the ticks used in the previous study was established with *O. erraticus* collected sometime 1970s and the 1990s when ASFV was circulating in the region. However, the conspecific ticks used in our study were from a colony started with specimens collected from the same region where ASF had been eradicated for 20 years and, therefore, co-evolutionary forces for the adaptation of ASFV to infect and persist in *O. erraticus* could have been weakened through time. The same observations have been published for mosquitoes, that is, the local genetic evolution of vector populations may influence pathogen replication, dissemination, and transmission [60–62].

Further studies assessing how the microbiome may influence the vector competence of *Ornithodoros* ticks for ASFV are needed given the recognition that the microbiota associated with ticks can shape their immunological response to infectious agents and determine the ability to transmit tick-borne pathogens [59, 63]. The application of soft tick genomics will help unveil the molecular basis of soft tick competence for ASFV. This needs to be coupled with the development of genetic markers in *Ornithodoros* ticks and ASFV to investigate hypotheses of local coevolution.

## Conclusion

The replication and dissemination patterns of ASFV in soft ticks were different between the five tick–virus combinations tested. Replication and disseminationwere were shown to be major determinants underlying successful horizontal and vertical transmission of the virus. Factors such as the *Ornithodoros* immune response against ASFV infection, the genetic background of ASFV and/or soft ticks, and the tick microbiome may also play a major role in vector competence. These research areas merit attention to enhance preparedness against ASF outbreaks where *Ornithodoros* ticks may be involved as ASFV vectors.

## Supplementary information

**Additional file 1: Table S1.** The number and the stage of ticks used for the study.

**Additional file 2: Figure S1.** Plasmid used for the standard curve for qPCR. Beta-actin primers are shown in cyan and the beta-actin probe in yellow. The ASFV-VP72 gene primers are shown in green and the ASFV-VP72 probe in red.

## Data Availability

All data generated or analyzed during this study are included in this published article.

## Abbreviations

ASF: African swine fever
ASFV: African swine fever virus
HAD_50_: Hemadsorbing dose 50%
OeG: *Ornithodoros erraticus*–Georgia2007/1
OeO: *Ornithodoros erraticus*–OurT88/1
OmG: *Ornithodoros moubata*–Georgia2007/1
OmL: *Ornithodoros moubata*–Liv13/33
SPF: Specific Pathogen-Free
