# Supplementary material for: Differential vector competence of Ornithodoros soft ticks for African swine fever virus: What if it involves more than just crossing organic barriers in ticks?
Source: Parasit Vectors. 2020 Dec 9;13:618. doi: 10.1186/s13071-020-04497-1 (PMC7725119; doi:10.1186/s13071-020-04497-1)
Supplement: Supplementary file 1 — Additional file 1: Table S1. The number and the stage of ticks used for the study. [file 13071_2020_4497_MOESM1_ESM.pdf]

Time PI (month) / Tick-virus pair	0	1	2[a]	3	6	8[a]	10	12	13[a]
OmL	F = 8<br>M = 6<br>N = 1	F = 7<br>M = 8<br>N = 0	F = 8<br>M = 7<br>N = 0	F = 7<br>M = 8<br>N = 0	ND	ND	ND	ND	ND
OmG	F = 5<br>M = 10<br>N = 0	F = 5<br>M = 10<br>N = 0	F = 2<br>M = 12<br>N = 1	F = 6<br>M = 9<br>N = 0	ND	ND	ND	ND	F = 8<br>M = 8<br>N = 0
OeO	F = 2<br>M = 2<br>N = 6	F = 11<br>M = 5<br>N = 0	F = 9<br>M = 4<br>N = 2	F = 9<br>M = 3<br>N = 3	F = 5<br>M = 5<br>N = 0	F = 5<br>M = 5<br>N = 0	F = 5<br>M = 5<br>N = 0	F = 5<br>M = 5<br>N = 0	ND
OeG	F = 1<br>M = 3<br>N = 6	F = 5<br>M = 5<br>N = 5	F = 6<br>M = 6<br>N = 3	F = 10<br>M = 3<br>N = 2	F = 5<br>M = 5<br>N = 0	F = 5<br>M = 5<br>N = 0	F = 5<br>M = 5<br>N = 0	F = 5<br>M = 5<br>N = 0	ND
OvZ	F = 0<br>M = 1<br>N = 10	F = 0<br>M = 0<br>N = 9	F = 3<br>M = 2<br>N = 5	F = 2<br>M = 0<br>N = 8	ND	ND	ND	ND	F = 7<br>M = 9<br>N = 2

Additional file 1: Table S1: Number and stage of ticks used for qPCR kinetics and viral titration. [a] : tick used for qPCR and also viral titration. F = female, M = male and N = nymph. ND = not done.
