# Supplementary material for: Differential vector competence of Ornithodoros soft ticks for African swine fever virus: What if it involves more than just crossing organic barriers in ticks?
Source: Parasit Vectors. 2020 Dec 9;13:618. doi: 10.1186/s13071-020-04497-1 (PMC7725119; doi:10.1186/s13071-020-04497-1)
Supplement: Supplementary file 2 — Additional file 2: Figure S1. Plasmid used for the standard curve for qPCR. Beta-actin primers are shown in cyan and the beta-actin probe in yellow. The ASFV-VP72 gene primers are shown in green and the ASFV-VP72 probe in red. [file 13071_2020_4497_MOESM2_ESM.pdf]

ATGTACCCCGGTATTGCCGACCGTATGCAGAAGGAAATCACTGCCCTGGCCCCATCCACGATGAAGATCAAGA
TCATCGCTCCCCCCGAGAGGAAGTACTCCGTCTGGATCGGTGGTTCCATCCTGGCCTCCCTGTCCACCTTCCAG
CAGATGTAAAAATGATCCGTTTAGGTTGATGGGCTTTGGTCATCGTGTATATAAAAACTACGATCCCCGTGCCG
CAGTACTTAAAGAAACCTGTAAAGAAGTATTAAAGGAACTCGGACAGCTAGAAAACAACCCACTCCTGCAAAT
AGCAATAGAATAACTGCTCATGGTATCAATCTTATCGATAAGTTTCCATCAAAGTTCTGCAGCTCTTACATACCC
TTCCACTACGGAGGCAATGCAATTAAAACCCCCGATGATCCGGGTGCGATGATGATTACCTTTGCTTTGAAGCC
ACGGGAGGAATACCAACCCAGTGGTCATATT
